# PROFILE OF OKLAHOMA CLIENTS REFERRED TO ADULT PROTECTIVE SERVICES FOR ALLEGED SELF-NEGLECT

**DOI:** 10.1093/geroni/igac059.2726

**Published:** 2022-12-20

**Authors:** Samantha Tuft, Farida Ejaz, Miriam Rose, Courtney Reynolds

**Affiliations:** Benjamin Rose Institute on Aging, Cleveland, Ohio, United States; Benjamin Rose Institute on Aging, Cleveland, Ohio, United States; Benjamin Rose Institute on Aging, Cleveland, Ohio, United States; Benjamin Rose Institute on Aging, Cleveland, Ohio, United States

## Abstract

Self-neglect is the most commonly reported allegation of abuse to Adult Protective Service (APS) agencies nationwide. Researchers collaborated with Oklahoma APS to understand characteristics of allegedly self-neglecting clients who were reported to APS (focus of this paper), and to subsequently refer a random sub-sample to home-and community-based services and track outcomes. Clients (n = 188) were interviewed by phone; data were collected on demographic and background characteristics, physical and mental health, social support, social isolation, and the impact of COVID-19 on them. Most clients were white, 18% were American Indian, and 14% were African American. Their ages ranged from 18–92 years, 63% were women, most were low-income ($12,000-$15,000 annually), and 60% lived alone. Many used public benefits such as Medicare (75%), Social Security, and Medicaid. Based on PHQ scores, about a third had moderate to severe symptoms of depression; and 27% scored similarly on anxiety. More than a third saw family and friends less often during the pandemic. APS administrative data was also obtained on these clients and preliminary analyses indicated that the 188 study participants had a total of 205 allegations of self-neglect prior to the study, with 76% of the allegations needing a referral for community services. During the intervention phase, however, only 31 clients had any type of abuse allegations with 24 being for self-neglect. About half needed community services. It is possible that services they were referred to prior and during the study was related to the decline in self-neglect allegations during the study period.

